# Comparative Analysis of Food Additives in Products with and Without Added Sugar on the Polish Market

**DOI:** 10.3390/foods15061046

**Published:** 2026-03-16

**Authors:** Aleksandra Kołodziejczyk, Justyna Nowak

**Affiliations:** 1 Faculty of Public Health in Bytom, Medical University of Silesia, 41-900 Bytom, Poland; d201294@365.sum.edu.pl; 2 Department of Metabolic Disease Prevention, Faculty of Public Health in Bytom, Medical University of Silesia, 41-902 Bytom, Poland

**Keywords:** food labeling, low-calorie sweeteners, food composition, product reformulation, consumer information, Central Europe, market survey

## Abstract

**Background/Objectives:** Limiting sugar intake is an important factor in preventing many diseases, which has led to growing interest in products labeled as “no added sugar”. Reformulation of food products may affect the types and number of additives used, highlighting the need for systematic analysis. The aim off this study was to compare the presence of additives in food products available on the Polish market, categorized as “with added sugar” or “no added sugar”. **Methods:** The analysis was based on label data from 1278 food products collected in the second and third quarters of 2023. Products were categorized according to sugar content, and the presence, types, and number of additives were compared between groups. **Results**: Products without added sugar generally had a simpler composition and contained fewer additives. The most frequently identified additive groups in products with sugar were acidity regulators and antioxidants, present several times more often than in sugar-free products. Preservatives, colors, emulsifiers, and other additives were also significantly more frequent in sweetened products, while sweeteners were more common in sugar-free products. The average number of additives per product was 1.6 for sugar-free products and 3.8 for products with added sugar. **Conclusions**: The results indicate that the presence and number of additives are closely related to whether a product contains added sugar. Products labeled as “no added sugar” tend to have simpler compositions and fewer additives, reflecting changes in recipes related to sugar reduction.

## 1. Introduction

Chronic diseases account for approximately 74% of deaths worldwide, placing a significant burden on households and healthcare systems [1]. One of the main factors contributing to the development of these diseases is lifestyle, including diet [1]. Excessive sugar consumption has a particularly well-documented negative impact on health [2,3]. Therefore, current guidelines recommend limiting sugar consumption as an important element of chronic disease prevention [2,4].

Growing concerns about the effects of sugar consumption have led to the implementation of systemic measures, such as educational campaigns, advertising restrictions, taxes on sweetened beverages, front-of-pack labeling, and food reformulation [5,6,7]. In response, manufacturers are increasingly taking steps to reduce sugar content while maintaining the sensory acceptability of their products [8]. Consequently, the number of products containing sugar substitutes on the market is growing [6].

Food additives, defined as substances intentionally introduced during processing, packaging, or storage, perform a range of sensory and technological functions, such as improving the color, flavor, shelf life, consistency, and stability of products [9,10]. Their presence can be observed in more than half of food products worldwide [11] and are consumed daily by billions of people [10]. Although their use is strictly regulated [12,13], for example, within the European Union under Regulation (EC) No 1333/2008 [12], excessive consumption can lead to adverse health effects [11,14,15], such as hematological disorders, allergic reactions, reproductive problems, autoimmune diseases, and an increased risk of certain cancers [14].

Food reformulation can involve both reducing the amount of added sugars and replacing them with various additives [5,16]. Consumers are increasingly interested in products labeled “sugar-free,” which, according to the current definition, do not contain mono- or disaccharides, nor any ingredients containing these sugars due to their sweetening properties [12]. Although they constitute an important element of food reformulation strategies, it should be noted that replacing sugar with low-calorie sweeteners does not fully reflect their actual health impact [17]. Highly processed products without added sugar may acquire a seemingly health-promoting image in the minds of consumers, which is not always justified by their actual composition [17].

Data regarding the composition of such products is still limited, highlighting the need for systematic comparative analyses. Previous studies have shown that products labeled “no added sugar” tend to have a lower energy value and significantly lower contents of saturated fatty acids, carbohydrates, and sugars, as well as a higher fiber content, compared with products containing added sugar, indicating that product reformulation may result in more favourable nutritional profiles [18]. In addition, analyses of low- and no-calorie sweeteners have indicated that sugar-free products are significantly more likely to contain polyols and other sweetener types than products with added sugar, suggesting that sugar reduction strategies are associated with substantial modifications in product formulation and may influence cumulative sweetener exposure in consumers [19]. At the same time, detailed data on the distribution and frequency of food additives in such products remain limited, which underscores the need for further systematic evaluation to determine whether reformulation strategies can support public health or may also introduce potential risks.

The aim of this study was to compare the presence and types of food additives in products available on the Polish market with and without added sugar. The study also aimed to assess whether products labelled as “no added sugar” differ in their additive profiles and whether they have a potentially more favourable composition.

## 2. Materials and Methods

### 2.1. Materials

The analysis included food products available on the Polish market, divided into two categories: products with added sugar and products clearly labeled “without added sugar.” The study sample consisted of a total of 1278 food products, of which 744 were “without added sugar” and 534 contained sugar. The category “without added sugar” included only products with this labeling and, in accordance with Regulation (EC) No 1333/2008 [12], did not contain added mono- or disaccharides, but could contain naturally occurring sugars, e.g., from fruit or milk. Products without this labeling, even if they did not contain added sugars, were not included in this category. The category “with added sugar” included products that contained added mono- or disaccharides beyond those naturally present.

Information on ingredients was collected in the second and third quarters of 2023 from product labels in three brick-and-mortar stores and four online stores. The points of sale were randomly selected and included both large nationwide retail chains and smaller, local outlets. This approach allowed for the inclusion of various food distribution channels and a wide range of brands and product categories available in the national market. For products offered at more than one point of sale, each was assigned a single database entry to avoid duplication and maintain consistency in the analysis. The analyzed products represented several food categories, including desserts, milk and dairy products, drinks, cereal flakes, breath fresheners, sweets and snacks, and vegetables, fruits and their products. The detailed distribution of the analyzed products across individual food categories and the complete product selection procedure have been described in previous publications concerning the same research group [18,19].

Product composition was recorded based on manufacturer label declarations. All data were entered into an MS Excel spreadsheet to create a structured research database. To ensure full anonymity, each product was assigned a unique identification number. These identifiers were assigned randomly, preventing any link between the results and a specific manufacturer or brand.

The study was observational and did not involve any interventions or experiments with human participants. Only publicly available data were analyzed. Therefore, approval from a Bioethics Committee was not required.

### 2.2. Methods

The study focused on comparing the labels of products labeled “without added sugar” with their sugar-containing counterparts. The formulation composition was analyzed, including both basic ingredients and additives used in production. The identified additives were grouped into the following functional categories: colorants (E100–E199), preservatives (E200–E299), acidity regulators and/or antioxidants (E300–E399), emulsifiers and/or stabilizers and/or gelling agents and/or thickeners (E400–E499), flavor enhancers (E600–E699), sweeteners (E950–E970), and other additives (E500–E599, E900–E949, E999–E1520).

The substances considered in the study were consistent with the EU list of additives approved for use in food, as set out in Annex II to Regulation (EC) No 1333/2008 of the European Parliament and of the Council of 16 December 2008 on food additives [12]. Each product was assessed for the presence of individual additives and the number of different additives used in the recipe of a single product. The analysis enabled the identification of both the types and the frequency of additives in the product groups studied. Furthermore, the ingredient profiles of “sugar-free” products were compared with their sugar-containing counterparts, allowing for the identification of differences in the use of additives.

### 2.3. Statistical Analysis

The data collected in the study were statistically analyzed using STATISTICA 13.3 (StatSoft Polska, Kraków, Poland). For qualitative variables, the frequencies of individual variants were calculated, presenting the results both as absolute values and percentages. Data distribution was assessed using the Shapiro-Wilk test to verify normality. Significance tests were used to assess differences between groups. Additionally, differences between the two structural indicators were analyzed to compare product categories. Relationships between variables were examined using the chi-square (Chi^2^) test. The significance level was α = 0.05. *p* values below this threshold were considered to indicate statistically significant differences. Results were presented in both tabular and graphical formats, facilitating a visual assessment of the observed relationships and trends.

## 3. Results

Based on the analysis of product composition, it was found that most food additive groups were significantly more frequently present in products containing sugar than in those without added sugar. In the analyzed product group, the most frequently identified additive categories were acidity regulators and antioxidants, with their presence observed several times more frequently in products containing sugar. The most frequently reported additives among all analyzed products were citric acid (E330) and lecithins (E322) (Supplementary Table S1). Citric acid was found in 11.2% of products without added sugar and 41.8% of products with added sugar. Lecithins, on the other hand, were detected in 8.9% of products without added sugar and 32.8% of products with added sugar.

Colors were less common, but their use was also significantly more frequent in sweetened products. Carotenes (E160a) were the most common color in both groups (4.0% of products without added sugar and 9.4% of products with added sugar, respectively). Preservatives were found in 10.3% of sugar-free products and 20% of sugar-containing products. The most common preservative in sugar-containing products was malic acid (E296) (8.2%), while in the sugar-free group, potassium sorbate (E202) was most common (5.8%). The results of the percentage comparison of products in terms of the presence of these groups are presented in Table 1.

Emulsifiers and/or stabilizers and/or gelling agents and/or thickeners were found significantly more frequently in the group of products with added sugar. Among the products without added sugar, gum arabic (E414) was the most frequently reported additive from this group (3.5%). In the products with added sugar, polyglycerol polyricinoleate (E476) accounted for the largest percentage (8.4%) of this additive group. Among the products analyzed, none of the products contained flavor enhancers. Among the group of sweeteners classified as food additives, sucralose (E955) was the most frequently used. It was found in 13.7% of the products without added sugar and in 10.7% of the products with added sugar. The presence of other additives was recorded in 15.2% of the products without added sugar and 30.5% of the products with added sugar. This difference was statistically significant. Modified starch (E1422) was the most frequently used additive in this group. It was found in 5.9% of products with no added sugar and in 12.0% of products with added sugar. The results of the percentage comparison in terms of the presence of the indicated groups are presented in Table 2.

A comparative analysis by established category revealed significant differences in the frequency of specific groups of additives between products with and without added sugar (Table 3). Colors were present in all product groups containing added sugar, while in sugar-free products they were not detected in milk and dairy products or cereals. The analysis revealed that desserts were the product category most frequently containing this group of additives. Colors were more frequently used in most categories of sweetened products than in products without added sugar.

Preservatives were most frequently used in beverages, with their share being significantly higher in sweetened products. Preservatives were also more frequently observed in sweets and snacks with added sugar, while the opposite trend was observed in desserts, where preservatives were more frequently found in sugar-free versions (*p* < 0.05). In the remaining categories, the differences in the percentage of products containing preservatives were not statistically significant.

Analysis of products by established categories revealed that each group contained products containing acidity regulators and/or antioxidants. Their presence was particularly prevalent in beverages and in sweets and snacks with added sugar, exceeding 90%. Significantly higher occurrences of these additives in sweetened products were also found in desserts, cereals, and the group of vegetables, fruits, and their products (*p* < 0.05).

The analysis of individual substances revealed differences in the dominant additives depending on the presence of sugar. In sugar-free products, sucralose was the most common additive in desserts (50.6%) and the group of vegetables, fruits, and their products (13%). In the groups of cereals (17.1%) and snacks and sweets (23.1%), maltitol was the most common additive, while in breath-freshening products, sorbitol was the most common additive (100%). In the group of milk and dairy products, citric acid (E330) (20%) and sodium citrates (E331) (20%) dominated.

In the group of sweetened products, the key additive was citric acid (E330), widely used in beverages (75.4%), desserts (43.5%), and vegetables, fruits, and their products (79%). Lecithins (E322) played a significant role in cereals (44.8%) and snacks and sweets (66.7%), while modified starch (E1401) played a significant role in dairy products (50%). Sucrose esters of fatty acids (E473) were the most common additives in breath-freshening products (22.2%).

A comparative analysis of products for sweeteners listed in Regulation (EC) No. 1333/2008 revealed their more frequent presence in products without added sugar. Additives from this group were found in all analyzed breath-freshening products without added sugar. However, they were not present at all in milk and milk products or breath-freshening products with added sugar. A significantly higher presence of sweeteners in products with added sugar was found only in the beverages and vegetables, fruits, and their products groups.

A comparison of products for the presence of emulsifiers and/or stabilizers and/or gelling agents and/or thickening agents in the products revealed their presence in most of the analyzed groups. Only cereals without added sugar did not contain additives from this group. The percentage of products containing emulsifiers and/or stabilizers and/or gelling and/or thickening agents was significantly more frequent (*p* < 0.05) in products with added sugar, especially in the groups of beverages, cereals, sweets and snacks, and vegetables, fruits, and their products. The exception was breath-freshening products, where a higher proportion of these additives was observed in sugar-free versions.

The remaining additives showed a similar trend to the previously analyzed additives. Their presence predominated in sweetened products, particularly desserts and dairy products. Sugar-free versions were characterized by a lower saturation level in this group of additives. The percentage of products containing other additives was significantly more frequent (*p* < 0.05) in products with added sugar, such as desserts, milk and dairy products, cereals, sweets, and snacks. However, in products without added sugar, the presence of other additives was not significantly more frequent.

A comparative analysis of the products in terms of the groups described is presented in Table 4.

**Figure 1:**
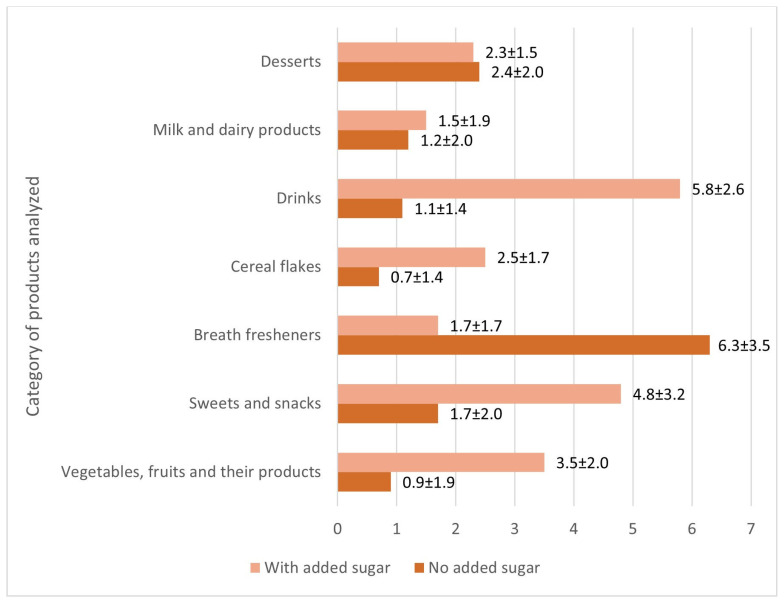
Comparison of the average number of additives used in one food product by category (data are presented as mean ± standard deviation).

A comparison of the average amounts of additives used in one group of analyzed food products showed that products with no added sugar contained an average of 1.6 additives per product, while products with added sugar contained 3.8. The highest number of additives was found in breath-freshening products. For products with no added sugar, a higher average amount of additives per product was observed in the desserts and breath-freshening groups compared to products with added sugar. A higher amount of additives in products with added sugar compared to products without added sugar was observed in the other product categories analyzed. The beverages group presented a significant discrepancy in the compared results, with products with added sugar having an average of 5.8 additives per product and products with no added sugar having 1.1. These results are presented in Figure 1.

**Figure 2:**
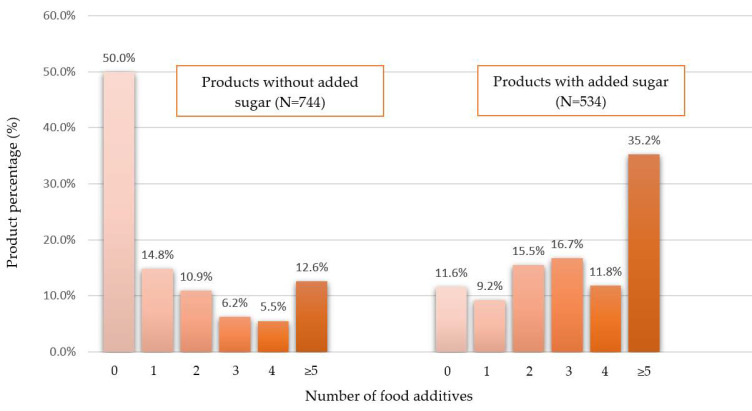
Percentage comparison of products in terms of the number of food additives per food product.

Analysis of the number of additives in food products revealed clear differences in the compositional structure between products containing sugar and their sugar-free counterparts. Figure 2 shows the number of food additives present in food products: a total of 50% of products without added sugar and 88.4% of products containing sugar contained at least one food additive. Products without added sugar most often contained no or a small number of additives, indicating a relatively simpler composition. In contrast, products with added sugar more often contained multiple additives.

## 4. Discussion

Consumers typically expect minimal processing and no additives in products labeled “organic” or “natural.” This is not always the case with foods labeled “sugar-free,” and many recipes contain chemical compounds that improve the sensory and technological quality of the products [20]. Although EU law clearly regulates the permissible use of food additives, the inability to monitor their total intake from various sources remains a real problem. This phenomenon poses a potential risk of exceeding safe levels and may result in adverse health effects [21,22].

Sugar consumption remains consistently high in Europe, particularly among children, with the largest amounts coming primarily from sweets and sweetened beverages, as confirmed by previous epidemiological analyses [22,23,24,25,26,27]. The average daily sugar consumption worldwide in 2023 was 197.54 kcal/person/day [28]. In Poland, despite a decline in unprocessed sugar consumption, its share in the overall diet is increasing [29]. Drinking sugar-sweetened beverages is associated with an increased risk of obesity, cardiovascular disease, metabolic syndrome, and type 2 diabetes [30,31,32,33,34]. A growing number of parents declare their willingness to limit their sugar intake, but the lack of clear information on labels hinders informed dietary choices [35]. In the light of international risk assessments and recommendations regarding the consumption of simple sugars, both the World Health Organization (WHO) and the European Food Safety Authority (EFSA) emphasize the need to limit their share in the diet [34,36,37]. Analyzing product composition is particularly important in the context of public health.

The study found that 50% of sugar-free products and 88.4% of sugar-added products contained additives, indicating significant differences between these categories. Similar trends for sugar-added products were also observed in international analyses. A study of the Brazilian market revealed the presence of additives in 79% of analyzed foods in 2021 [38] and 84% of products in 2025 [39]. In Iran, additives were identified in 95.3% of food products intended for children [40]. In Hong Kong, additives were most commonly found in confectionery products (91.1%) [41]. In the Brazilian market, they dominated in fruit drinks, carbonated drinks, and sweetened products (over 60% of products in each group) [38], and in France, in over 85% of products in groups such as artificially sweetened beverages, ice cream, industrial sandwiches, biscuits, and cakes [42]. The similarity of the obtained results confirms the global nature of additive use. It should also be emphasized that products labeled “without added sugar” were characterized by a significantly lower presence of additives, which may be the result of formula reformulation.

Regardless of the category, the most commonly used additives were citric acid (E330) and lecithin (E322). Citric acid was present in the recipes of 10.9% of sugar-free products and 41.8% of sugar-added products. Lecithin was detected in 8.9% of sugar-free products and 32.8% of sugar-added products. In France and Brazil, these substances were also among the most frequently reported additives [42,43]. The frequent use of these additives in various product categories highlights their role as standard functional additives in the food industry and indicates the repeatability of food formulation practices at the international level.

Color plays a key role in food appeal, and its loss during storage or processing often leads manufacturers to use colorants [44]. Analysis of the presence of colorants in the tested products revealed the absence of these substances in most products without added sugar (85.1%) and with added sugar (70.8%). In an analysis of the Brazilian market, covering a wide range of food products, 72.2% of items did not contain colorants [42], which was consistent with our results. The use of synthetic colorants continues to increase in food processing, particularly in the production of beverages and confectionery, due to their low price and color stability [45]. Excessive consumption of synthetic colorants, especially by children, may pose a health risk, as emphasized by previous studies [46]. In our own studies, the most common colorant found in products was carotenes, which belong to the group of natural pigments. The presence of natural pigments in the analyzed products therefore appears to be beneficial, but further analysis of their health impact seems necessary.

Analysis of the presence of preservatives in the studied products showed that most products did not contain these types of additives, but their use was more common in products with added sugar, particularly in beverages, which are a significant source of sugar in the diet [24,47]. In studies conducted on the Brazilian market, preservatives were detected in almost one-third of the products, similarly to those in Poland, suggesting that their presence is not specific to one region or type of food [38]. The lower frequency of preservative use in sugar-free products suggests that changing recipes may effectively reduce the demand for these substances. Although additives from this group play an important role in inhibiting chemical transformations [48], studies suggest that artificial preservatives, like other artificial additives, may pose a health risk when consumed in excess, which prompts producers to look for natural alternatives [49,50,51,52,53]. Among products with added sugar, the most commonly used preservative was a natural substance, malic acid (E296), while in sugar-free products, the synthetic preservative potassium sorbate (E202) predominated. The dominance of different types of substances in both groups illustrates the varied selection of preservatives depending on the product profile. 

Antioxidants constitute another important group of technological additives, whose primary function is to limit oxidative processes, thus extending the shelf life of food products [54]. Although their use is considered safe when adhering to acceptable intake standards, they still raise consumer concerns, particularly in the case of synthetic compounds, which are chosen due to their easy availability and low application costs [55,56]. The analysis showed that the group of antioxidants and/or acidity regulators was more frequently found in products containing sugar. In the Hong Kong market, in a study covering a general sample of food products by category, acidity regulators were detected in 46.4% of confectionery products, with citric acid being the most frequently identified compound (39.4%) [41]. The similarity of the above results with our own analysis, in which citric acid was found in 41.8% of products with added sugar, indicates its universal technological significance in various market contexts. Citric acid was significantly less frequently used in sugar-free products (10.9%), yet it should be noted that it was the most frequently used ingredient in the study group. The use of a natural additive may indicate a positive trend, but it should be remembered that natural origin does not guarantee complete safety [55,56,57], justifying the need for further monitoring of the use of these substances.

The increase in the consumption of highly processed foods leads to an increased use of technological additives [58], including emulsifiers, stabilizers, gelling agents, and thickeners, which are referred to as food hydrocolloids [59]. These substances are present in food products in varying proportions. Our own analysis showed that most food products did not contain these substances, but they were more frequently present in the group of products with added sugar compared to the group of products without added sugar, which may be due to the higher technological requirements of these products. Due to the potential for adverse reactions associated with the consumption of certain additives [60], differences in their use between product groups may be important for further research on the function of additives in produced food. 

The next group of technological additives analyzed were flavor enhancers, also known as flavor potentiators, which are intended to intensify taste sensations [61]. The results of our study showed that none of the products, both “sugar-free” (N = 744) and those with added sugar (N = 534), contained this type of substance. The absence of flavor enhancers may be due to their limited effectiveness in dairy and sweet products, as indicated in the literature [61]. The observed lack of use of flavor enhancers in the studied sample reflects both the technological requirements of the products and the production practices resulting from the selection of ingredients. Flavor enhancers may have different effects depending on their consumption [62], which makes their presence in food an important aspect when analyzing the profile of technological additives.

Sweeteners were an important element of the discussed issue, and their detailed characteristics in relation to the analyzed product groups have already been presented in more detail [63]. This analysis confirmed that sweeteners were significantly more common in sugar-free products (33.9%), while their presence was also noted in 20% of products that already contained a sweetener in the form of sugar. This phenomenon indicates that sweeteners do not always act solely as a sugar substitute but can also be used to improve other aspects. In research conducted on the Hong Kong market, sweeteners were most frequently identified in confectionery products (18.9%), snacks (16.3%), and beverages (10.5%) [41]. Comparing these data with the results obtained in Poland, the particularly high frequency of their occurrence in beverages is noteworthy, both in sugar-free (25.7%) and sugar-containing (52.2%) variants. Such significant differences may be a consequence not only of differing consumer preferences and manufacturer strategies but also of the scope of the analyses—in Poland, only sweetened beverages were studied, while in Hong Kong, the entire beverage category was analyzed [41].

An analysis of the number of additives in the food products studied on the Polish market revealed that products with added sugar had a higher average number of additives than their sugar-free counterparts. The highest values were recorded in sweetened beverages (5.8 on average), indicating that the processing of this category promotes the more frequent use of many additives. A similar relationship was confirmed for breath-freshening products without added sugar (5.3), while cereals without added sugar had the lowest number of additives (0.7), which is due to their simpler formulation. Similar ranges were observed in other national studies—in pasteurized ready-made meals, the number of additives ranged from 0.9 in “leczo” (lecho) to 6.2 in “regionally inspired” products [63]. These results indicate a certain consistency in the distribution of additives despite differences in the type of products studied. Different results were noted in Hong Kong, where the average number of additives ranged from 0.3 in eggs and egg products to 4.2 in bread and bakery products [41]. Lower values in some categories result from the fact that the Hong Kong study also included natural products, such as eggs, which do not require additives. However, it should be emphasized that in all the indicated analyses, products with the highest number of additives exceeded four compounds on average, confirming the trend of their increased use in selected food products. Referring to similar groups analyzed in studies, the category of confectionery products analyzed in Hong Kong should be particularly noted. The average number of additives per product in this group was 4.0 [41]. In our study, products with added sugar had a similar average number of additives, amounting to 3.8. Differences were noted, among others, in dairy products, where the results were higher in Hong Kong (2.35 compared to 1.2–1.5 in Poland). However, the greatest discrepancies were found in sweetened beverages, where the average number of additives in Poland was as high as 5.8 compared to 1.6 in Hong Kong [41]. For snacks, the values in Poland were also higher (4.8 compared to 3.28), which could be due to the inclusion of sweets in the Polish analysis [41]. These results indicate that, despite local differences in composition, there is a clear trend towards the use of multiple food additives, which may be important for their cumulative consumption.

Analysis of the number of additives present in individual products revealed clear differences between the “no added sugar” and sugar-added categories. In Poland, sugar-free products rarely contained four or more additives (18.1%), while in sugar-added products this percentage was 47%. International studies also observed the widespread use of several additives in processed products, although the analyses did not distinguish between sugar-added and sugar-free products. In Hong Kong, 38.6–46.5% of products in selected categories contained four or more additives [30], in Brazil, 48% [38], and in France, 16.6% [42], with the lower French result more consistent with the Polish group of sugar-free products. However, it should be noted that the French study covered all products available on the market, including water, fats, and meat products, which results in a lower average number of additives [42]. At the same time, the lower presence of additives in “no added sugar” products in Poland reflects formulation changes resulting from product reformulation, leading to a simpler product composition.

Summarizing the results, the analysis clearly demonstrated that food products labeled “no added sugar” are characterized by simpler composition and fewer additives compared to their sugar-containing counterparts. It should be emphasized that the presence of additives alone does not pose a risk; what matters is their quantity and frequency of consumption [64]. In the context of a daily diet, “no added sugar” products can be a beneficial alternative, especially for those seeking to reduce their intake of sugar and excess additives, provided they are consumed as part of a balanced diet. These results emphasize the importance of informed dietary choices and label analysis in consumer decision-making.

## 5. Study Limitations

The combination of analyses conducted in brick-and-mortar stores and online platforms was a significant advantage of our study, providing a broad and up-to-date insight into product composition. However, this study had several significant limitations that may affect the interpretation of the obtained results. First, the analysis was based solely on information declared on product labels, which assumes their accuracy, although errors or inaccuracies in labeling cannot be ruled out. Furthermore, the study did not include the quantities of individual additives, which prevents the assessment of actual consumer exposure and comparison of potential intake levels with applicable safety standards. Accurately determining concentrations would require the use of various analytical methods and laboratory tests on a large number of samples, which was beyond the scope of this study. Another limitation is the single-year nature of the study, which does not allow for the consideration of seasonal fluctuations in product availability or changes in formulations over time. Repeating the analyses in subsequent years could provide a more comprehensive picture of market trends and changes in product composition. In addition, multiple comparisons were conducted across additive categories and product groups using chi-square tests without applying a formal correction for multiple testing. This aspect should be taken into account when interpreting statistically significant differences.

## 6. Conclusions

The comparative analysis showed that the presence and number of additives in food products in Poland are closely related to the product category “with added sugar” versus “no added sugar.” Products labeled as “no added sugar” had a simpler composition and were less likely to contain four or more additives. These findings may reflect broader industry trends aimed at simplifying product formulations in response to consumer demand for shorter ingredient lists.

The results suggest that manufacturers’ efforts to reduce sugar may also contribute to a lower number of additives. This observation may be relevant for future product development strategies and for initiatives promoting food reformulation. It is important to emphasize that food products can have diverse compositions, and making informed nutritional decisions based on label analysis remains crucial for consumers.

Including “no added sugar” products in the daily diet can be part of a strategy to improve nutritional quality, provided that a balanced diet is maintained. At the same time, it should be clearly stated that this study does not evaluate the direct impact of additives on human health. The findings are limited to the frequency and presence of additives in products, without quantifying consumer exposure or assessing potential health effects.

Nevertheless, systematic monitoring of product composition and reformulation provides important reference value for consumers, producers, and public health decision-makers. Future research should focus on monitoring changes in product formulations over time and on assessing the quantitative content of additives in order to better estimate consumer exposure and evaluate potential health implications. Continuous market monitoring may provide valuable information for both consumers and policymakers involved in nutrition and public health strategies. In addition, expanding similar analyses to other food categories and conducting comparative studies across different markets could contribute to a more comprehensive understanding of current trends in food product reformulation and additive use.

## Figures and Tables

**Table 1:** Percentage comparison of products in terms of the presence of selected groups of additives.

	Presence of Colorant	*p*	Presence of Preservatives	*p*	Presence of Acidity Regulators and/or Antioxidants	*p*
Products without added sugar (N = 744)	13.0%	<0.0001	10.3%	<0.0001	26.7%	<0.0001
Products with added sugar (N = 534)	29.2%		20.0%		74.3%	

**Table 2:** Percentage comparison of products in terms of the presence of selected groups of additives.

	Presence of Emulsifiers and/or Stabilizers and/or Gelling Agents and/or Thickening Agents	*p*	Presence of Flavor Enhancers	Presence of Sweeteners	*p*	Presence of Other Additives	*p*
Products without added sugar (N = 744)	15.9%	<0.0001	0%	33.9%	<0.0001	15.2%	<0.0001
Products with added sugar (N = 534)	35.8%		0%	20.0%		30.5%	

**Table 3:** Comparison of products in terms of individual groups of additives, divided into established categories.

Product Group		Presence of Colorant	*p*	Presence of Preservatives	*p*	Presence of Acidity Regulators and/or Antioxidants	*p*
Desserts	No added sugar (N = 81)	48.1% (N = 39)	0.063	8.6% (N = 7)	0.0408	29.6% (N = 24)	0.0116
	With added sugar (N = 46)	65.2% (N = 30)		0% (N = 0)		52.2% (N = 24)	
Milk and dairy products	No added sugar (N = 10)	0% (N = 0)	0.0456	0% (N = 0)	-	20% (N = 2)	0.5188
	With added sugar (N = 64)	29.7% (N = 19)		0% (N = 0)		12.5% (N = 8)	
Drinks	No added sugar (N = 109)	29.4% (N = 32)	0.0351	32.1% (N = 35)	<0.0001	45% (N = 49)	<0.0001
	With added sugar (N = 69)	44.9% (N = 31)		63.8% (N = 44)		97.1% (N = 67)	
Cereal flakes	No added sugar (N = 35)	0% (N = 0)	0.006	0% (N = 0)	0.1122	14.3% (N = 5)	<0.0001
	With added sugar (N = 58)	19% (N = 11)		6.9% (N = 4)		74.1% (N = 43)	
Breath fresheners	No added sugar (N = 8)	37.5% (N = 3)	0.4895	0% (N = 0)	0.3314	62.5% (N = 5)	0.0919
	With added sugar (N = 9)	22.2% (N = 2)		11.1% (N = 1)		22.2% (N = 2)	
Sweets and snacks	No added sugar (N = 255)	9% (N = 23)	<0.0001	2% (N = 5)	<0.0001	33.7% (N = 86)	<0.0001
	With added sugar (N = 207)	27.1% (N = 56)		19.8% (N = 41)		90.3% (N = 187)	
Vegetables, fruits and their products	No added sugar (N = 246)	5.7% (N = 14)	0.3556	12.2% (N = 30)	0.0503	12.6% (N = 31)	<0.0001
	With added sugar (N = 81)	8.6% (N = 7)		21% (N = 17)		81.5% (N = 66)	

**Table 4:** Comparison of products in terms of individual groups of additives, divided into established categories.

Product Group		Presence of Sweeteners	*p*	Presence of Emulsifiers and/or Stabilizers and/or Gelling Agents and/or Thickening Agents	*p*	Presence of Other Additives	*p*
Desserts	No added sugar (N = 81)	63.0% (N = 51)	<0.0001	22.2% (N = 18)	0.0533	33.3% (N = 27)	<0.0001
	With added sugar (N = 46)	2.2% (N = 1)		8.7% (N = 4)		100% (N = 46)	
Milk and dairy products	No added sugar (N = 10)	0% (N = 0)	-	30% (N = 3)	0.8219	10% (N = 1)	<0.0001
	With added sugar (N = 64)	0% (N = 0)		26.6% (N = 17)		100% (N = 64)	
Drinks	No added sugar (N = 109)	25.7% (N = 28)	0.0003	23.9% (N = 26)	0.0486	6.4% (N = 7)	0.8712
	With added sugar (N = 69)	52.2% (N = 36)		37.7% (N = 26)		5.8% (N = 4)	
Cereal flakes	No added sugar (N = 35)	28.6% (N = 10)	<0.0001	0% (N = 0)	0.0039	2.9% (N = 1)	0.0165
	With added sugar (N = 58)	0% (N = 0)		20.7% (N = 12)		20.7% (N = 12)	
Breath fresheners	No added sugar (N = 8)	100% (N = 8)	<0.0001	75% (N = 6)	0.0295	12.5% (N = 1)	0.9287
	With added sugar (N = 9)	0% (N = 0)		22.2% (N = 2)		11.1% (N = 1)	
Sweets and snacks	No added sugar (N = 255)	42.7% (N = 109)	<0.0001	21.2% (N = 54)	<0.0001	16.5% (N = 42)	<0.0001
	With added sugar (N = 207)	4.1% (N = 33)		45.4% (N = 94)		46.4% (N = 96)	
Vegetables, fruits and their products	No added sugar (N = 246)	14.2% (N = 35)	0.0008	5.3% (N = 13)	<0.0001	14.2% (N = 35)	0.8937
	With added sugar (N = 81)	30.9% (N = 25)		44.4% (N = 36)		14.8% (N = 12)	

## Data Availability

The data supporting the results of this study are available from the corresponding author upon reasonable request.

## Supplementary Materials

The following supporting information can be downloaded at: https://www.mdpi.com/article/10.3390/foods15061046/s1, Table S1: Frequency of individual food additives identified in the analyzed products.
