# Supplementary material for: Comparative Analysis of Food Additives in Products with and Without Added Sugar on the Polish Market
Source: Foods. 2026 Mar 16;15(6):1046. doi: 10.3390/foods15061046 (PMC13024859; doi:10.3390/foods15061046)
Supplement: Supplementary file 1 [file foods-15-01046-s001.zip › foods-4177660-supplementary.pdf]

foods

MDPI

Supplementary Table S1. Frequency of individual food additives identified in the analyzed products.

Additive	Products without added sugar (N=744)	Products with added sugar (N=534)
Curcumin (E100)	11 (1.5%)	17 (3.2%)
Riboflavins (E101)	18 (2.4%)	7 (1.3%)
Tartrazine (E102)	0 (0.0%)	2 (0.4%)
Sunset Yellow FCF (E110)	0 (0.0%)	1 (0.2%)
Carmine (E120)	4 (0.5%)	14 (2.6%)
Allura Red AC (E129)	0 (0.0%)	4 (0.7%)
Patent Blue V (E131)	0 (0.0%)	2 (0.4%)
Indigotine (E132)	0 (0.0%)	4 (0.7%)
Brilliant Blue FCF (E133)	7 (0.9%)	10 (1.9%)
Chlorophyllin (E140)	4 (0.5%)	17 (3.2%)
Copper complexes of chlorophylls and chlorophyllins (E141)	0 (0.0%)	15 (2.8%)
Caramels (E150a-d)	27 (3.6%)	38 (7.1%)
Vegetable Carbon (E153)	2 (0.3%)	3 (0.6%)
Carotenes (E160a)	30 (4.0%)	50 (9.4%)
Annatto (E160b)	2 (0.3%)	0 (0.0%)
Paprika extract (E160c)	0 (0.0%)	7 (1.3%)
Beta-apo-8′-carotenal (E160e)	0 (0.0%)	1 (0.2%)
Lutein (E161b)	0 (0.0%)	2 (0.4%)
Betanin (E162)	5 (0.7%)	7 (1.3%)
Anthocyanins (E163)	16 (2.2%)	12 (2.2%)
Calcium Carbonate (E170)	8 (1.1%)	11 (2.1%)
Titanium Dioxide (E171)	0 (0.0%)	2 (0.4%)
Sorbic Acid (E200)	3 (0.4%)	0 (0.0%)
Potassium Sorbate (E202)	43 (5.8%)	19 (3.6%)
Benzoic Acid (E210)	2 (0.3%)	0 (0.0%)
Sodium Benzoate (E211)	8 (1.1%)	13 (2.4%)
Sulfur Dioxide (E220)	2 (0.3%)	6 (1.1%)
Sodium metabisulphite (E223)	0 (0.0%)	3 (0.6%)
Lactic Acid (E270)	2 (0.3%)	18 (3.4%)
Carbon Dioxide (E290)	32 (4.3%)	38 (7.1%)
Malic acid (E296)	6 (0.8%)	44 (8.2%)
Ascorbic Acid (E300)	27 (3.6%)	33 (6.2%)
Tocopherol (E306)	0 (0.0%)	1 (0.2%)
Alpha-Tocopherol (E307)	2 (0.3%)	24 (4.5%)
BHA (E320)	2 (0.3%)	0 (0.0%)
BHT (E321)	0 (0.0%)	0 (0.0%)
Lecithins (E322)	66 (8.9%)	175 (32.8%)
Sodium Lactate (E325)	0 (0.0%)	3 (0.6%)
Calcium Lactate (E327)	1 (0.1%)	2 (0.4%)
Citric Acid (E330)	83 (11.2%)	223 (41.8%)
Sodium Citrates (E331)	28 (3.8%)	49 (9.2%)
Potassium Citrates (E332)	0 (0.0%)	5 (0.9%)
Calcium Citrates (E333)	2 (0.3%)	0 (0.0%)
Tartaric Acid (E334)	1 (0.1%)	2 (0.4%)
Phosphoric Acid (E338)	4 (0.5%)	4 (0.7%)

Sodium Phosphates (E339)	1 (0.1%)	9 (1.7%)
Potassium Phosphate (E340)	5 (0.7%)	9 (1.7%)
Tricalcium Phosphate (E341)	2 (0.3%)	1 (0.2%)
Sodium malates (E350)	0 (0.0%)	4 (0.7%)
Sodium Alginate (E401)	1 (0.1%)	2 (0.4%)
Agar (E406)	13 (1.7%)	2 (0.4%)
Carrageenan (E407)	5 (0.7%)	11 (2.1%)
Carob Gum (E410)	2 (0.3%)	11 (2.1%)
Guar Gum (E412)	8 (1.1%)	21 (3.9%)
Gum Arabic (E414)	26 (3.5%)	26 (4.9%)
Xanthan Gum (E415)	10 (1.3%)	1 (0.2%)
Gellan Gum (E418)	10 (1.3%)	11 (2.1%)
Sorbitols (E420)	12 (1.6%)	2 (0.4%)
Mannitol (E421)	1 (0.1%)	0 (0%)
Glycerol (E422)	11 (1.5%)	21 (3.9%)
Pectins (E440)	29 (3.9%)	70 (13.1%)
Ammonium phosphatides (E442)	0 (0.0%)	9 (1.7%)
Sucrose Acetate Isobutyrate (E444)	3 (0.4%)	1 (0.2%)
Glycerol Esters of Rosin (E445)	7 (0.9%)	11 (2.1%)
Diphosphates (E450)	1 (0.1%)	14 (2.6%)
Sodium Tripolyphosphate (E451i)	1 (0.1%)	0 (0.0%)
Cellulose Gum (E466)	5 (0.7%)	1 (0.2%)
Magnesium Salts of Fatty Acids (E470b)	9 (1.2%)	1 (0.2%)
Mono- and Diglycerides of Fatty Acids (E471)	9 (1.2%)	29 (5.4%)
Sucrose Esters of Fatty Acids (E473)	0 (0.0%)	3 (0.6%)
Polyglycerol polyricinoleate (E476)	0 (0.0%)	45 (8.4%)
Sodium Stearoul-2-lactylate (E481)	0 (0.0%)	2 (0.4%)
Sodium Carbonate (E500)	24 (3.2%)	53 (9.9%)
Sodium Bicarbonate (E500II)	13 (1.7%)	17 (3.2%)
Potassium Carbonate (E501)	3 (0.4%)	8 (1.5%)
Ammonium Carbonates (E503)	11 (1.5%)	37 (6.9%)
Ammonium Bicarbonates (E503ii)	0 (0.0%)	6 (1.1%)
Magnesium Carbonates (E504)	3 (0.4%)	5 (0.9%)
Calcium Chloride (E509)	12 (1.6%)	0 (0.0%)
Sodium Hydroxide (E524)	0 (0.0%)	4 (0.7%)
Calcium Citrates (E533)	3 (0.4%)	5 (0.9%)
Silicon Dioxide (E551)	4 (0.5%)	0 (0.0%)
Beeswax (E901)	0 (0.0%)	17 (3.2%)
Carnauba Wax (E903)	5 (0.7%)	2 (0.4%)
Shellac (E904)	0 (0.0%)	5 (0.9%)
Acesulfame K (E950)	34 (4.6%)	9 (1.7%)
Aspartame (E951)	23 (3.1%)	0 (0.0%)
Cyclamic acid and its Na and Ca salts (E952)	0 (0.0%)	2 (0.4%)
Isomalt (E953)	10 (1.3%)	2 (0.4%)
Saccharin and its Na, K and Ca salts (E954)	0 (0.0%)	4 (0.7%)
Sucralose (E955)	102 (13.7%)	57 (10.7%)
Steviol glycosides (E960)	31 (4.2%)	13 (2.4%)
Neotame (E961)	1 (0.1%)	0 (0.0%)
Maltitols (E965)	2 (0.3%)	5 (0.9%)
Xylitol (E967)	27 (3.6%)	0 (0.0%)
Erythritol (E968)	62 (8.3%)	1 (0.2%)
Polydextrose (E1200)	0 (0.0%)	3 (0.6%)
Modified Starch (E1401)	44 (5.9%)	64 (12.0%)

https://doi.org/10.3390/foods15061046

Sodium Starch Octenylsuccinate (E1450)	1 (0.1%)	0 (0.0%)
Propylene Glycol (E1520)	20 (2.7%)	0 (0.0%)

https://doi.org/10.3390/foods15061046
